# The complete chloroplast genome of *Aruncus dioicus* var. *kamtschaticus* (Rosaceae)

**DOI:** 10.1080/23802359.2021.1906173

**Published:** 2021-03-28

**Authors:** Hwa-Jung Suh, Juhyeon Min, Jongsun Park, Sang-Hun Oh

**Affiliations:** a Department of Biology, Daejeon University, Daejeon, Korea; b InfoBoss Inc, Seoul, Republic of Korea; c InfoBoss Research Center, Seoul, Republic of Korea

**Keywords:** *Aruncus*, chloroplast genome, Korea, Rosaceae

## Abstract

*Aruncus dioicus* var. *kamtschaticus* is an economically important herb in the cold temperate regions of East Asia, and displays highly variable morphological features. Completed chloroplast genome of *A. dioicus* var. *kamtschaticus* isolated in Korea is 157,859 bp long with four subregions: 85,972 bp of large single copy and 19,185 bp of small single-copy regions separated by 26,351 bp of inverted repeat regions. The genome includes 131 genes (86 protein-coding genes, eight *rRNA*s, and 37 *tRNA*s). Phylogenetic analyses show that our chloroplast genome was clustered with two partial chloroplast genomes of *A. dioicus*.

*Aruncus dioicus* (Walter) Fernald is one of the three species of *Aruncus* L., broadly distributed in the temperate regions of the Northern Hemisphere from central Europe and the Caucasus to the Himalayas, China, Korea, Japan, Russia (Ussuri, Sakhalin, Kamchatka, and Kurile Islands), and North America (Ohwi 1965; Czerepanov 1995; Kim H and Ju 1997; Cuizhi and Alexander 2003; Lee 2006; Shetekauri and Jacoby 2009; Mellichamp 2015). It is a large perennial herb up to 3 m tall with estipulate, pinnately compound leaves, loose panicles with many small, unisexual flowers, and pendulous, follicular fruits. *Aruncus dioicus* var. *kamstchaticus* (Maxim.) H. Hara 1955 is a common East Asian variety, found in mixed forests at high elevation (Ohwi 1965; Cuizhi and Alexander 2003). In Korea, it has been traditionally used for foods (Chung et al. 2010) and for medicinal and cosmetic usages (Kim M-S et al. 2011; Youn et al. 2012; Zhang Q and Kim 2014). It shows a wide range of morphological variations, resulting in taxonomic confusion (Hara 1957; Cuizhi and Alexander 2003). The complete chloroplast genome will be helpful to understand the origin of the economically important plants.

Total DNA was extracted from fresh leaves collected on Mt. Hwangbyeong, Gangwon-do, Republic of Korea (37°42′49.03′′N, 128°42′28.39′′E; voucher number: *Suh 7282* in Daejeon University Herbarium (TUT)) by using DNeasy Plant Mini Kit (QIAGEN, Hilden, Germany). Sequencing library was constructed using Illumina TruSeq Nano DNA Library Preparation Kit (Illumina, San Diego, CA) following manufacturer’s recommendations with around 350-bp DNA fragments. 1.99-Gbp raw sequences were obtained using HiSeqX at Macrogen Inc., Korea, and filtered by Trimmomatic version 0.33 (Bolger et al. 2014). Chloroplast genome was *de novo* assembled with Velvet version 1.2.10 (Zerbino and Birney 2008) and base-pair confirmation was performed by SOAPGapCloser version 1.12, BWA version 0.7.17, and SAMtools version 1.9 (Li et al. 2009; Li 2013). All processes were conducted under the environment of Genome Information System (GeIS; http://geis.infoboss.co.kr). Geneious R11 version 11.0.5 (Biomatters Ltd., Auckland, New Zealand) was used for annotation based on *A. dioicus* chloroplast (KY419942).

Chloroplast genome of *A. dioicus* var*. kamtschaticus* (GenBank accession is MW115132) is 157,859 bp long (GC ratio is 36.4%) and has four subregions: 85,972 bp of large single copy (LSC; 34.2%) and 19,185 bp of small single copy (SSC; 30.0%) regions are separated by 26,351 bp of inverted repeat (IR; 42.4%). It contains 131 genes (86 protein-coding genes, eight *rRNA*s, and 37 *tRNA*s); 19 genes (eight protein-coding gene, four *rRNA*s, and seven *tRNA*s) are duplicated in IR regions. No structural variation was found among the genomes of the species of tribe Spiraeeae examined.

Two partial chloroplasts (KY419932 and KY419926) were used for identifying intraspecific variations: 180 SNPs and 69 INDELs against KY419932 and 299 SNPs and 84 INDELs against KY419926 in LSC, SSC, and IRb regions. Numbers of intraspecific variations are relatively large based on the intraspecific variation analysis of chloroplast genomes (Park, Xi, et al. 2020). High levels of intraspecific variations were found in many plant species, such as some species in Orchidaceae (Oh et al. 2019a, 2019b; Park, Suh, et al. 2020; Kang et al. 2020) and Rosaceae (Cho et al. 2019; Heo et al. 2020), *Camellia japonica* (Park et al. 2019), *Euscaphis japonica* (Oh and Park 2020), *Selaginella tamariscina* (Park, Kim, et al. 2020), and *Marchantia polymorpha* subsp. *ruderalis* (Kwon et al. 2019).

Thirteen chloroplast genomes to represent the major lineages of tribe Spiraeeae (Potter et al. 2007) were used in phylogenetic analysis of the maximum likelihood (ML) and Bayesian inference (BI). Seventy-eight genes of LSC, SSC, and IRb regions were included in the analyses. A heuristic search was used with nearest-neighbor interchange branch swapping, the Tamura-Nei model, and uniform rates among sites to construct ML phylogenetic tree with default values for other options using MEGA X (Kumar et al. 2018). Bootstrap analysis with 1000 pseudoreplicates was also conducted. BI tree was constructed by MrBayes version 3.2.7a (Ronquist et al. 2012). The GTR model with gamma rates was used as a molecular model. A Markov-chain Monte Carlo (MCMC) algorithm was employed for 10,000,000 generations, sampling trees every 200 generations, with four chains running simultaneously.

The phylogenetic tree shows that *A. dioicus* var*. kamtschaticus* clustered with two previously sequenced chloroplasts of *A. dioicus* forming a strongly supported clade (Figure 1). Our new chloroplast genome sequences are determined from the Korean sample. Geographic origin of the two *A. dioicus* published chloroplast genomes (Zhang SD et al. 2017) is unknown, but it is unlikely to be from Korea. The high level of intraspecific variation suggests that the chloroplast genome can be utilized to understand phylogeographic pattern of *A*. *dioicus* across the whole distribution range as well as relationships among the several varieties of the species.

**Figure 1.**
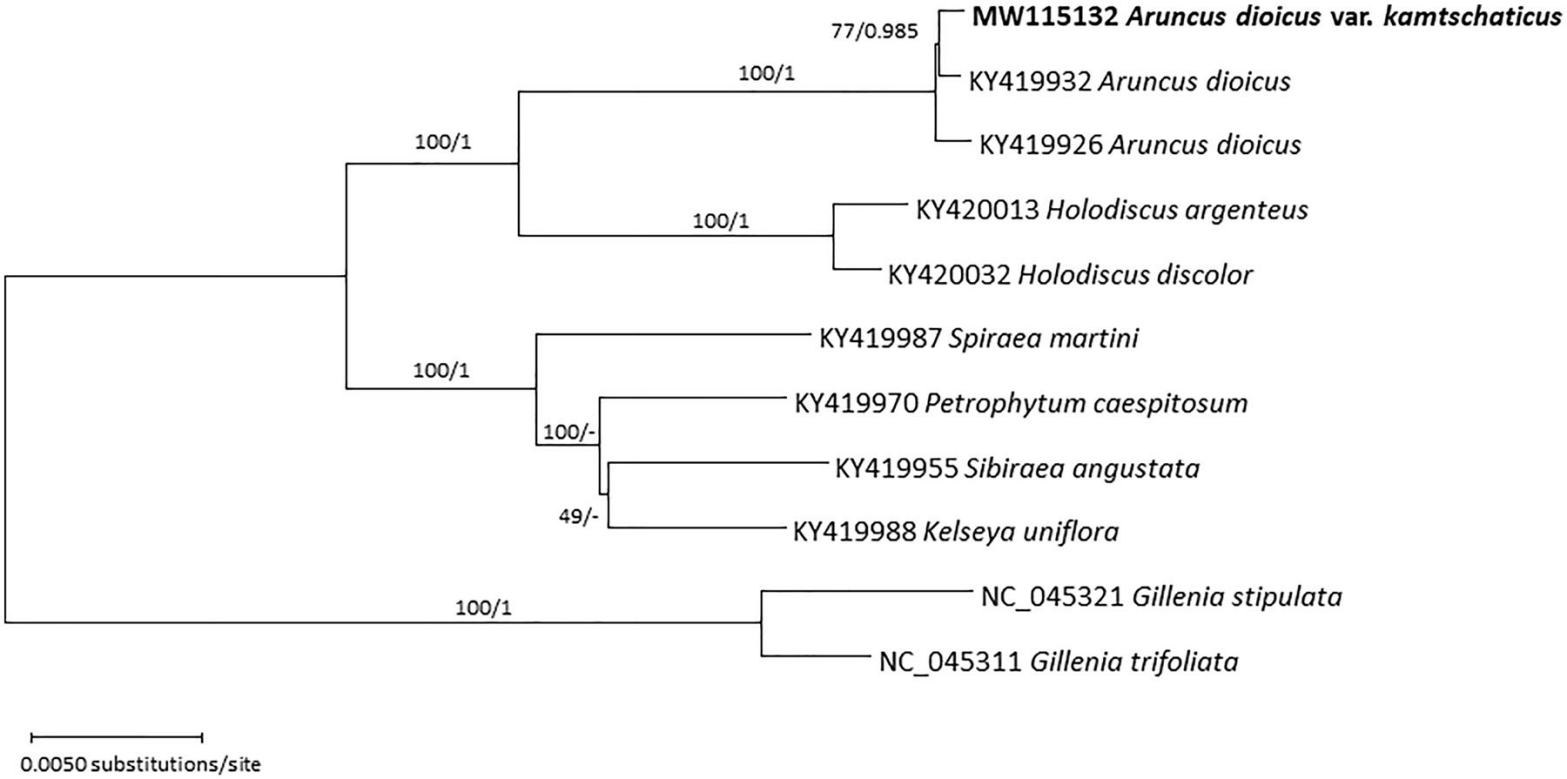
Maximum likelihood phylogenetic trees of ten chloroplast genomes of seven species of tribe Spiraaeae with two *Gillenia* species as outgroups. Phylogenetic tree was drawn based on maximum likelihood tree. The numbers above branches indicate bootstrap support values of maximum likelihood and posterior probability from the BI inference, respectively.

## Data Availability

Chloroplast genome sequence can be accessed *via* accession number of MW115132 in GenBank of NCBI at https://www.ncbi.nlm.nih.gov. The associated BioProject, SRA, and Bio-Sample numbers are PRJNA668550, SAMN16414964, and SRR12807287, respectively.
